# Analysis of prognostic factors in soft tissue sarcoma: Cancer registry from a single tertiary hospital in Indonesia. A retrospective cohort study

**DOI:** 10.1016/j.amsu.2020.07.053

**Published:** 2020-08-07

**Authors:** Ferdiansyah Mahyudin, Mouli Edward, Muhammad Hardian Basuki, Yunus Basrewan, Kukuh Dwiputra Hernugrahanto, Adhinanda Gema Wahyudiputra

**Affiliations:** Department of Orthopedics & Traumatology, Faculty of Medicine, Universitas Airlangga / Dr. Soetomo General Hospital, Jl. Rungkut Mapan FD-2, Surabaya, East Java, Indonesia

**Keywords:** Distant metastasis, Local recurrence, Modified glasgow prognostic score, Neutrophils/lymphocytes ratio, Overall survival, Soft tissue sarcoma

## Abstract

**Background:**

Soft tissue sarcoma is one cause of mortality in adult malignancies. This tumor is rare, persistent, and highly-recurrent. Many patients are came in late stage. It is important to identify a prognostic tool that is reliable, easily obtainable, and widely applicable. The aim of this study is to investigate and analyze the prognostic value of clinicopathological and biomarker factors in patients with soft tissue sarcoma.

**Methods:**

This retrospective study extracts data from the musculoskeletal tumor registry from January 2012 to December 2018 in a single tertiary hospital. Eighty patients with diagnosis of soft tissue sarcoma were included. Preoperative modified Glasgow Prognostic Score, Neutrophils/Lymphocytes Ratio, Hemoglobin, serum lactate dehydrogenase data were analyzed along with demographic, clinical, radiological and histopathological data. The relationship between variables on overall survival, distant metastasis, and local recurrence were evaluated using univariate and multivariate Cox regression.

**Results:**

On univariate analysis, there was significant relationship between hemoglobin, Neutrophils/Lymphocytes Ratio and modified Glasgow Prognostic Score with overall survival (p = 0.031, HR = 1.99; p = 0.04, HR = 1.129; and p = 0.044, HR = 3.89). A significant relationship was found between age and soft tissue sarcoma stage with distant metastasis (p = 0.046, HR = 1.95; and p = 0.00, HR = 3.22). In addition, we also found significant relationship between surgical margin with local recurrence (p = 0.018, OR = 3.44). However, on multivariate analysis the independent prognostic factor for overall survival was only modified Glasgow Prognostic Score (HR = 2.138; p = 0.011). Stage IIIA (HR = 5.32; p = 0.005) and IIIB (HR = 13.48; p = 0.00) were independent prognostic for distant metastasis. Surgical margin was independently associated with local recurrence (HR = 14.84; p = 0.001).

**Conclusion:**

Modified Glasgow Prognostic Score can be used as prognostic tool of overall survival in soft tissue sarcoma patients. Moreover, stage of STS and surgical margin can be used as a prognostic factor for distant metastasis and local recurrence of soft tissue sarcoma respectively.

## Highlights

• Study about prognostic tool of STS in emergent country is still limited.
• Inflammatory process and biomarkers have important roles in STS progression.
• mGPS can be used as simple prognostic tool of overall survival in STS patients.
• Surgical margin and STS stage have prognostic role in recurrence and metastasis.

## 1 Introduction

Soft tissue sarcoma (STS) is a rare and persistent malignant tumor originating in mesenchymal tissue of any organs except bones and cartilage. It constitutes only 1–2% of malignancies in adults. About 75% of the tumor involves the extremities [1,2]. Data from a national referral hospital for cancer in Indonesia showed that STS is among the ten most common cancer cases in 2013 [3]. Unfortunately, most patients come late for diagnosis [1,2]. Despite adequate treatment protocol, patients with high-grade STS are still at risk for recurrence and distant metastasis. In the United States it is estimated that in 2020 there will be 13.130 STS cases with a five-year survival rate of 16–81% [4,5].

Since fifteen years ago, prognostic tools for STS have been limited to the use of clinical and histopathological factors such as age at the time of diagnosis, tumor size, histological grade and subtype, tumor depth and location, and tumor margin [6,7]. Therefore, it is important to identify a prognostic tool that is reliable, easily obtainable, and widely applicable to improve the ability of risk stratification and to help with treatment guideline.

Previous studies showed that inflammatory process and its biomarkers have been known to play important roles in STS progression [8,9]. The haemoglobin level and some biomarkers, like c-reactive protein (CRP), Neutrophil/Lymphocyte ratio (NLR), and C-Reactive Protein/Albumin ratio (CAR), could serve as promising prognostic factors [[10], [11], [12], [13]]. Another study on modified Glasgow Prognostic Score (mGPS) using combination of albumin and CRP showed that mGPS could predict the outcome in cancer cases although it did not specifically predict those of distant metastasis and local recurrence [11]. To our best knowledge, the studies to analyze the prognostic factors of STS, especially in emergent nations, are still limited. The aim of this study is to investigate and analyze prognostic value of clinicopathological and biomarker factors in patients with STS.

## 2 Methods

### 2.1 Study design

This is a retrospective study. Between January 2, 2012 to December 30, 2018, 106 patients with diagnosis of STS at Dr. Soetomo General Academic Hospital, a tertiary hospital in Indonesia, were recorded. All patients were registered in the musculoskeletal tumor registry. This registry collects patients’ demographic data; preoperative, intraoperative, and postoperative data that include laboratory, radiological, and histopathological examination; treatment protocol; and postoperative follow-up including any complication or mortalities that may occur. The diagnosis and treatment protocols of STS in all patients were discussed and decided in a Clinico-pathological Conference (CPC) between pathologists, radiologists, oncologist, and orthopaedic surgeons.

### 2.2 Clinical data collection

The inclusion criteria were all patients with diagnosis of STS that had followed the hospital's treatment protocol with minimum follow-up of one year. Patients with incomplete registry, failure to comply with treatment and follow-up protocol, metastasis prior to treatment, and history of blood disorders, such as anemia, thalassemia, or other similar abnormality were excluded from the study. Out of 106 patients, only 80 patients were eligible for the study (Fig. 1). Independent variables in this study were age, tumor size, cancer stage, tumor anatomy location, tumor type, hemoglobin, NLR, mGPS, serum lactate dehydrogenase (LDH), type of therapy and surgical margin.

**Flow Chart 1:**
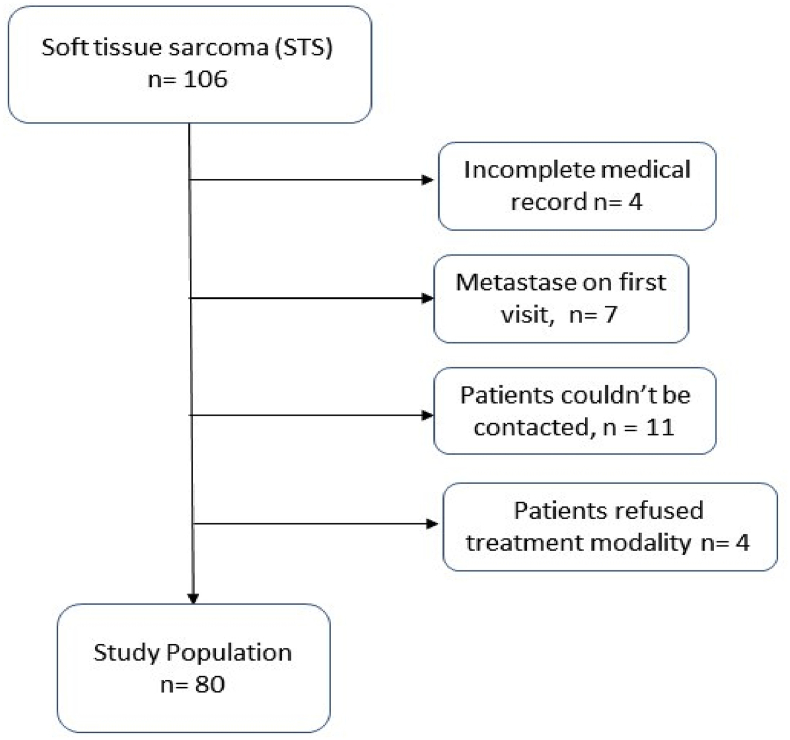
The process of determining the study population.

The laboratory data were taken preoperatively one to three days before the surgery was performed. Haemoglobin level were divided into three categories: (1) low (<13 mg/dL male, <11 mg/dL female), (2) normal (13–16 mg/dL male, 11–14 mg/dL female) and (3) high (>16 mg/dL male and> 14 mg/dL in women). The mGPS was classified using the value of CRP and albumin. Patients were scored 2 if they had an increase in CRP (>1.0 mg/dL) and a low albumin (<3.5 g/dL); scored 1 with increased CRP alone and scored 0 with normal CRP [10]. The NLR were obtained from the ratio of the total number of neutrophils to lymphocytes measured from an analysis of complete blood count. NLR was high if the value is ≥ 5 [14]. LDH level was high if the values > 200 IU/L [14].

Clinicopathological data were tumor size, histopathological diagnosis, tumor stage and surgical margin. Tumor size was defined as the maximum diameter of the tumor obtained from MRI examination. Histopathological examination was performed and confirmed by two senior consultants of musculoskeletal pathologist. STS tumor types followed the latest WHO's 2013 classification. Tumor stages were divided into four: I, II, III, IV, in accordance with the system of the American Joint Committee on Cancer Staging (AJCC) and the 7th edition of the International Union Against Cancer (IUCC). Surgical margins were divided into two based on R+1 mm classification: specimens with tumor within 1 mm of the inked border were categorized as microscopically positive (R1), and R0 if there was at least 1 mm of healthy tissue from the inked border [15]. All of the patients underwent therapy in accordance with the STS treatment protocol at the hospital. The treatments were either wide excision or radical excision, with or without chemotherapy or radiotherapy (Table 1). Inoperable patients were treated with chemotherapy and radiotherapy. This study had received ethical approval by the hospital's ethical committee.

**Table 1:** Characteristics of STS patients.

		n	Percentage (%)
Age	<20 years	11	13.8
	20–40 years	27	33.8
	40–60 years	33	41.3
	>60 years	9	11.3
Tumor Location	Shoulder	4	5.0
	Forearm	12	15.0
	Hand	4	5.0
	Thigh	38	47.5
	Leg	14	17.5
	Pelvic	4	5.0
	Arm	3	3.8
	Back	1	1.3
Tumor Stage	Stage II	10	12.5
	Stage IIIA	21	26.3
	Stage IIIB	49	61.3
Tumor type	*Synovial Sarcoma*	10	12.5
	*Rhabdomyosarcoma*	4	5.0
	*Undifferentiated pleomorphic sarcoma*	19	23.8
	*Extraskeletal Osteosarcoma*	2	2.5
	*Phosphaturic mesenchymal tumor*	1	1.3
	*Leiomyosarcoma*	2	2.5
	*Undifferentiated round cell sarcoma*	4	5.0
	*Myxofibrosarkoma*	3	3.8
	*Malignant peripheral nerve sheath tumor(MPNST)*	5	6.3
	*liposarcoma dedifferentiated*	3	3.8
	*Desmoplastic small round cell tumor*	1	1.3
	*Adult Fibrosarcoma*	7	8.8
	*Low grade fibromyxoid sarcoma*	2	2.5
	*Extraskeletal mesenchymal chondrosarcoma*	1	1.3
	*Malignant solitary fibrous tumor (malignant SFT)*	2	2.5
	*Embryonal rhabdomyosarcoma*	2	2.5
	*Undifferentiated spindle cell sarcoma*	9	11.3
	*Malignant variant of diffuse type tenosynovial giant cell tumor*	1	1.3
	*Myxoid liposarcoma*	1	1.3
	*Extraskeletal myxoid chondrosarcoma*	1	1.3
Tumor Size	<5 cm	9	11.3
	5–10 cm	21	26.3
	>10 cm	50	62.5
Type of Therapy	Amputation (A)	11	13.8
	Amputation Chemotherapy (AK)	8	10.0
	Amputation Chemotherapy Radiotherapy (AKR)	4	5.0
	Amputation Radiotherapy (AR)	2	2.5
	*Wide Excision (W)*	20	25.0
	*Wide Excision* Chemotherapy (WK)	13	16.3
	*Wide Excision* Radiotherapy Chemotherapy (WKR)	11	13.8
	*Wide Excision* Radiotherapy (WR)	8	10.0
	Chemotherapy Radiotherapy (KR)	3	3.8

n: number.

### 2.3 Statistical analysis

The end-points for univariate and multivariate analyses were overall survival (OS), local recurrence (LR), and distant metastasis (DM). Overall survival (OS) time was calculated from the date of the diagnosis to the date of death regardless of any cause. Deaths up to December 2019 were included. Local recurrence (LR) was defined as recurrence of the same histological type of sarcoma in the same region of previous tumor, and confirmed by biopsy. Distant metastasis (DM) was defined as the cancer that had spread from the original (primary) tumor to distant organs or distant lymph nodes. The study were presented in line with the STROCSS criteria [16]. Univariate survival analysis was done using Kaplan-Meier survival method and log rank test for statistical significance. A two-sided value of p < 0.05 was referred to as significant. Multivariate analysis was calculated using Cox Proportional hazard regression model with stepwise backward procedure. Only significant variables in univariate analysis that were included in the multivariate analysis. All statistical analysis used SPSS 23 software (SPSS Inc., Chicago, IL, USA).

## 3 Results

There were 46 male (57.5%) and 34 female (42.5%) patients. The mean and median age of the patients at the time of diagnosis was 41.69 ± 18.16 and 42 years. Majority of the patients were in 40–60 years old (41.3%) followed with the age group of 20–40 years old (3.8%). The mean follow-up time was 32 months (range 13–71 months). During the follow-up period, 23.5% patients died, 33.8% developed local recurrence, and 21.2% developed DM. The median OS was 32 months.

More than 60% anatomic site of the tumor were in lower extremity. Undifferentiated round cell sarcoma was the most common tumor type found in this study (23.8%). More than 50% patients came in stage IIIB. More than 60% of the patients had lesions >10 cm. More than 60% patients had wide excision as the surgical procedure. Based on our study there were 2 out of 42 patients with soft tissue sarcoma in femoral and pelvic region that had bone marrow violation. The characteristics of tumor location, stage, histological diagnoses, size, and therapy modalities are shown in Table 1. Thirty six patients (45%) scored normal mGPS while the other 28 (35%) and 16 (20%) patients scored mGPS score of 1 and 2 respectively. Thirty three patients scored high NLR (41.25%). Thirty eight patients had high LDH level (47.5%). Forty seven patients were anemia (58.75%). Mean values of NLR, LDH, haemoglobin level were 5.4 ± 18.16, 245.41 ± 188.69, and 11.75 ± 2.23 respectively. Based on surgical margin, the proportion of patients with R0 and R1 categories was 72.5% (58 patients) and 27.5% (22 patients) respectively. Table 2 shows the results of univariate cox regression tests based on OS, DM, and local recurrence. There were significant associations in mGPS (p = 0.044), NLR (p = 0.04), and Hb level (p = 0.031) with OS. The other variables were not significant. There were significant associations between age and tumor stage with DM, with a p-value of 0.046 and 0.00 respectively.

**Table 2:** The relationship between clinicopathological variables with distant metastasis, overall survival and local recurrence (Univariate analysis).

Characteristics	Variable	Metastasis (M)		Survival(S)		Recurrence (R)		HR			p-value		
		Yes	No	alive	death	Yes	No	M	S	R	M	S	R
Age	<20	4	7	8	3	3	8	1.95	0.304	1.158	0.046^a^	0.551	0.952
	20–40	7	20	21	6	11	16						
	40–60	6	28	28	6	7	27						
	>0	0	8	4	4	5	3						
Tumor Size	<5 cm	1	9	8	2	2	8	0.000	1.230	0.813	0.999	0.811	0.804
	5–10 cm	5	16	16	5	9	12						
	>10 cm	11	38	37	12	15	34						
Tumor Stage	Stage II	1	9	8	2	2	8	3.22	1.444	1.375	0.000^a^	0.667	0.655
	Stage IIIA	5	16	16	5	9	12						
	Stage IIIB	11	38	37	12	15	34						
Tumor Location	Shoulder	0	4	3	1	2	2	1.083	1.082	3.000	0.293	0.642	0.361
	Forearm	3	9	9	3	4	8						
	Hand	1	4	5	0	2	3						
	Thigh	10	27	28	9	12	25						
	Leg	1	13	11	3	6	8						
	Pelvic	0	4	3	1	0	4						
	Arm	1	1	0	2	0	2						
	Back	1	1	2	0	0	2						
mGPS	mGPS 0	6	29	28	7	13	22	0.600	3.890	1.696	0.414	0.044^a^	0.433
	mGPS 1	8	21	23	6	9	20						
	mGPS 2	3	13	10	6	4	12						
NLR	≤5	11	36	38	9	19	28	0.522	1.120	1.054	0.244	0.040^a^	0.392
	>5	6	27	23	10	7	26						
LDH	Low	11	31	34	8	15	27	1.005	1.000	1.002	0.058	0.811	0.178
	High	6	32	27	11	11	27						
Hb	Low	7	40	31	16	15	32	2.781	1.99	0.656	0.220	0.031^a^	0.582
	Normal	9	22	29	2	10	21						
	High	1	1	1	1	1	1						
Surgical Margin	R0	44	14	46	12	15	43	2.015	1.789	3.440	0.312	0.300	0.018^a^
	R1	19	3	15	7	12	10						

mGPS: modified Glasgow Prognostic Score; NLR: Neutrophil Lymphocyte Ratio; LDH: Lactate Dehydrogenase; Hb: Hemoglobin; M: metastasis, S: Survival, R: recurrence.

a Statistically significant.

There was a significant difference in surgical margin with LR (p = 0.018, HR = 3.44). There was no significant relationship in univariate analysis between modality of therapy with OS (p = 0.698), DM (p = 0.415) and LR (p = 0.467).

**Fig. 2:**
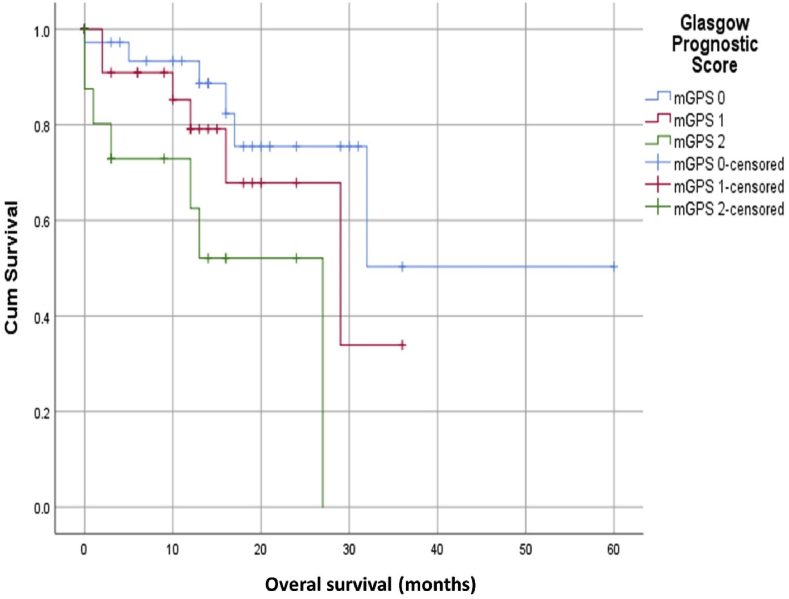
The relationship between mGPS and overall survival in patients with Soft Tissue Sarcoma.

The log rank test for mGPS on OS Kaplan Meier curve (Fig. 2) was significant (p = 0.018). Patients with mGPS 0 had overall one-year and three-year survival rates of 93.3% and 50.3% respectively. Lower overall one-year and three-year survival rates (85.2% and 33% respectively) were observed in patients with mGPS 1. Patients with mGPS 2 had the lowest one-year OS rate of only 52%.

Multivariate analysis showed that mGPS was the only independent prognostic factor for OS in soft tissue sarcoma patients (HR = 2.138; CI = 1.187–3.851). Stage IIIA (HR = 5.32; CI = 1.65–17.0) and IIIB (HR = 13.48; CI = 4.45–40.95) were the independent prognostic factor for DM. Moreover surgical margin was an independent prognostic factor for LR in our study (HR = 14.84; CI = 3.20–68.30) (Table 3).

**Table 3:** The relationship between variables and overall survival, distant metastasis and local recurrence.

Multivariate analysis	Variable	*Hazard Ratio* (HR)	95% CI	P value
Overall survival	Low Hb	0.889	0.70–1.12	0.327
	High NLR	1.027	0.96–1.10	0.439
	High mGPS	2.138	1.187–3.851	0.011^a^
Distant Metastasis	Age (Older)	1.01	0.99–1.02	0.206
	Stage IIIA	5.32	1.65–17.00	0.005^a^
	Stage IIIB	13.48	4.45–40.95	0.000^a^
Local Reccurence	Surgical margin R1	14.84	3.20–68.30	0.001^a^

a Statistically significant.

## 4 Discussion

There was a significant relationship between age and DM in STS (p = 0.046, HR = 1,95). This might be due to decreasing immunological conditions in older patients with tumor, thus making it easier for tumors to spread to other locations. This result is relevant with the study of Trovik et al. where age was associated with local metastasis and recurrence [7]. Older patients have metastasis more often than younger patients. In older patient, more cell DNA are damaged [17]. Patients with old age will generally have worse outcomes because they often come with high stage tumors and less aggressive local and systemic treatment [18]. However, in this study, age was not related to STS mortality (p = 0.551). This is in conjunction with the study conducted by Maretty, that age had no effect on mortality from soft tissue sarcoma^.^ The study said that age had more significant impact on LR [19].

Three previous studies before stated that an increase in NLR was significantly correlated with poor outcomes [8,20,21]. It was consistent with the results of this study which found a significant relationship between NLR values with OS (p = 0.04, HR = 1.129). The actual mechanism of the prognostic impact of NLR for patients with STS is still unclear. High NLR was associated with elderly patient (≥65 years), tumor size (>5 cm), tumor depth, high grade, and high TNM stage (III-IV) [20]. Tumor ability to progress is not only depend on tumor's intrinsic characteristic but also on the tumor microenvironment. The high value of NLR shows an increase in neutrophil response to tumors. Neutrophil infiltration can suppress the immune activity of lymphocytes and Natural Killer cells by producing chemokines and cytokines [20]. Neutrophils also stimulate tumor angiogenesis by producing proangiogenic factors, such as growth factors, chemokines and proteases, that trigger tumor progression [22,23]. The study of Ji et al. in a mouse model showed that tumor cells secrete chemotactic cytokines, inflammatory proteins, and interleukins to attract neutrophils in tumor microenvironment. The high density of tumor tissue infiltrated with neutrophils provides a favorable environment for cancer development by removing many inflammatory mediators such as tumor necrosis factor a (TNF-a), vascular endothelial growth factor (VEGF), interleukin-2 (IL-2), interleukin-6 (IL-6), and interleukin-10 (IL-10) [24,25]. Low lymphocyte count might reflect inadequate immune response [20]. Lymphocytes themselves have a role in the cytotoxic cells death and production of cytokines that inhibit the proliferation and migration of tumor [26,27]. NLR shows a balance between tumor pro-inflammatory and anti-tumoral immune status. An increase in NLR in malignant patients indicates greater inflammatory-tumor condition and tumor necrosis, thus giving a poor outcome [21].

Preoperative anemia has been found as a prognostic factor of poor outcome for patients with soft tissue sarcoma [13,28]. There was a significant relationship between Hb and OS (p = 0.031, HR = 1.99). Based on the study conducted by Szkandera et al. low hemoglobin level significantly affects patient's condition. Our study found similarity which low hemoglobin levels were more common in patients who did not survive, while the surviving patients had higher levels. Furthermore in their study, majority of anemia were found in patients with concomitant worse prognosis factor like high tumor grade, deep tumor, bigger tumor size, and older age. The cause of anemia in cancer cases can be due to dysfunction of iron metabolism, inadequate production of erythropoetin, and inadequate response of the bone marrow to erythropoetin. Low Hb levels are also associated with poor tumor oxygenation of up to 50–60% of tumors. In advanced cases, it can show hypoxic tissue area. Hypoxia in tumors is associated with malignant development in terms of recurrence, loco-regional spread and metastasis [13,29].

Lactate dehydrogenase (LDH) is known to describe how much influence the tumor has on the body. It becomes a significant prognostic factor in several types of malignancies. LDH is released from various organs and tissues when cells are attacked by neoplasms [30]. Nevertheless, in this study, there was no significant relationship of LDH values with OS (p = 0.332). This could be due to the fact that not all of the tumor cells produce LDH, but only a few, so that the description of LDH levels did not have a significant relationship with mortality in patients due to tumors. This was consistent with the study by Nakamura et al. that LDH values were not related to survival in patients with soft tissue sarcomas [31].

No significant relationship was found between modality of therapy with OS, LR, and DM. This could be caused by the presence of other more determining factors such as tumor stage, age and surgical margin. The most common treatment was wide excision (25%) followed by combination of wide excision and chemotherapy (16.3%). Inoperable STS patients undergo chemotherapy and radiotherapy alone without surgery. Our inoperable patients came with a high-staged tumor in pelvis or spine with size of more than 8 cm. This was consistent according to the National Comprehensive Cancer Network (NCCN) guidelines in which unresectable STS were treated with radiotherapy, chemotherapy or regional limb therapy [32]. There were no specific modification for neo-adjuvant chemo-radiation therapy in patient with tumor identified near to bone marrow like upper thigh. The neo-adjuvant chemotherapy used in this study was Doxorubicin and Ifosfamide.

From the multivariate analysis, mGPS was the only independent factor with OS in STS patient. CRP is a marker of inflammatory processes. As the tumor growth, large amounts of inflammatory cytokines, especially interleukins-1 and -6 are produced to stimulate hepatocytes to produce more CRP. In addition to being a biomarker for inflammation, CRP also functions as an important cytokine for cellular and biological processes in host defense. Study by Nakamura in 2013 involving 332 adult patients with primary soft tissue sarcoma showed that CRP values were significantly associated with oncological outcomes and patient survival [33]. Not only do albumin levels reflect the systemic inflammatory response but also the amount of lean tissue in the host. Albumin levels also reflect the nutritional and functional status of the patient [11]. In this study, the result related to mGPS (p = 0,011, HR = 2,13) was in accordance with study by Maretty et al.which found the relationship between NLR, mGPS, and Aarhus Composite Biomarker Score (ACBS) with mortality. Spence et al. in their study also stated that mGPS is an easy, inexpensive, and sensitive score system for predicting outcome or mortality in STS patients [8,11,34].

The majority of patients in this study were in stage IIIB (61.3%). One-year mortality in patients with stage IIIB was the greatest with 63.15%. Thus, on multivariate analysis, STS stage was the only independent factor associated with the occurrence of DM. The higher the stage results in the higher risk for metastasis (p = 0.005, HR = 5.32 for IIIA; and p = 0.00, HR = 13.48 for stage IIIB). This result is similar to the majority of studies that staging classification of tumors is a significant prognostic factor for mortality, metastatic events and LR [[35], [36], [37], [38]]. Higher stage was associated with an increase in tumor size, high histological levels based on FNCLCC grading, and the presence of nodules [39].

Patient with surgical margin R1 had significantly higher risk of LR of STS (p = 0.001, HR = 14.84). This was consistent with the study of Liu et al. and Stefanovski et al. which said that the surgical margin can be a predictive factor for recurrence [38,40]. If the distance of the surgical margin free from the tumor is greater, the recurrence rate will be lower. This might happen because patients with high stage have a higher risk in their surgery to not obtain the tumor-free edge of the incision. Positive surgical margins were a strong prognostic factor of LR in patients with STS in extremities. Microscopic positive margins were associated with high LR and low disease free survival rates [41]. It is still debatable what minimum margin distance required to reduce the risk of LR of high grade STS [42]. The AJCC margin classification simply stated margins as negative (R0), microscopically positive (R1), or grossly positive (R2). A recommendation from NCCN stated that adjuvant radiotherapy was given to patients with soft tissue sarcomas with resection of less than 1 cm from the tumor since the minimum margin spacing needed to reduce the risk of LR of high-grade soft-tissue sarcoma remains undefined. Nevertheless, the last study by Cates et al. show that the Musculoskeletal Tumor Society and margin distance classifications gave more predictive value for LR of high-grade pleomorphic soft tissue sarcomas than AJCC R system.They stated that adequate margin spacing was defined as ≥1 mm which require adjunctive radiation therapy, whereas that of ≥5 mm requires no adjunctive radiation therapy[43]. Therefore, based on the results of this study, a healthy tissue on surgical margin of at least 1 mm is suggested (R0) to reduce the risk of local recurrence.

Studies on the prognostic factor of STS are still rare in developing countries with limited diagnosis and treatment modalities. Prognostic factors that have been studied in developed countries so far have not been applied in Indonesia so that this research can help to find solution for prognostic factors that are more appropriate and practical to use. However, this study has several limitations. Firstly, the sample size was not considerably large enough. Based on our observation, this might be due to low reported case of STS because of lack of public awareness to STS. Furthermore, this lack of public awareness results in patients coming to hospitals only after the tumor become unbearable, not to mention those unreported case with fatality. Secondly, this is a retrospective study with a minimum follow-up of one year. We are aware that the recurrence would occur in the first five years. Unfortunately, large number of patients failed to comply with the follow-up program. For instance, some preferred to discontinue the treatment and prefer alternative non-medical treatment. Thirdly, it is a single-center study which might be subjected to bias. To improve the outcome of the study, it is suggested to conduct a multi-center study with prospective design and larger sample size.

## 5 Conclusion

Modified Glasgow Prognostic Score can be used as a simple, reliable, and practical prognostic factor of OS in patients with soft tissue sarcoma (STS). The higher the AJCC stage of STS (stage III) remain the higher risk of DM in STS. Moreover, surgical margins of R0 (less than 1 mm) can be consider as margin of safety for decreasing the risk of local recurrence in soft tissue sarcoma cases.

## Funding/support statement

This research did not receive any specific grant from funding agencies in the public, commercial, or not-for-profit sectors.

## Provenance and peer review

Not commissioned, externally peer reviewed.

## Declaration of competing interest

The authors declare no conflict of interest in this study.
